# Abdomen/pelvis computed tomography in staging of pediatric Hodgkin Lymphoma: is it always necessary?

**DOI:** 10.1002/cam4.829

**Published:** 2016-08-03

**Authors:** Piero Farruggia, Giuseppe Puccio, Alessandra Sala, Alessandra Todesco, Monica Terenziani, Rosamaria Mura, Salvatore D'Amico, Tommaso Casini, Clara Mosa, Marta Pillon, Maria Paola Boaro, Gaetano Bottigliero, Roberta Burnelli, Caterina Consarino, Fausto Fedeli, Maurizio Mascarin, Katia Perruccio, Elisabetta Schiavello, Angela Trizzino, Umberto Ficola, Alberto Garaventa, Mario Rossello

**Affiliations:** ^1^ Pediatric Hematology and Oncology UnitOncology DepartmentA.R.N.A.S. Ospedali CivicoDi Cristina e BenfratelliPalermoItaly; ^2^ Department of Sciences for Health Promotion and Mother and Child CareUniversity of PalermoPalermoItaly; ^3^ Clinica PediatricaUniversita' Milano – BicoccaA.O. San GerardoFondazione Monza e Brianza per il bambino e la sua mamma (MBBM)MonzaItaly; ^4^ Dipartimento di Oncoematologia PediatricaUniversità di PadovaPadovaItaly; ^5^ Pediatric Oncology UnitFondazione IRCCS Istituto Nazionale dei TumoriMilanoItaly; ^6^ Pediatric Hematology‐OncologyOspedale Pediatrico MicrocitemicoCagliariItaly; ^7^ Oncologia PediatricaClinica PediatricaCataniaItaly; ^8^ Dipartimento di Oncoematologia PediatricaA.O.U MeyerFirenzeItaly; ^9^ Dipartimento di Pediatria II Ateneo di NapoliServizio di Oncologia PediatricaNapoliItaly; ^10^ Oncoematologia PediatricaAzienda Ospedaliera UniversitariaOspedale Sant'AnnaFerraraItaly; ^11^ Pediatric Hemato‐oncology UnitDipartimento di Onco‐EmatologiaCatanzaro HospitalCatanzaroItaly; ^12^ Department of PediatricsNiguarda Ca' Granda HospitalMilanoItaly; ^13^ S.S. Radioterapia Pediatrica e Area GiovaniIRCCSCentro di Riferimento Oncologico AvianoPordenoneItaly; ^14^ Oncoematologia PediatricaAzienda Ospedaliera‐Universitaria di PerugiaPerugiaItaly; ^15^ Dipartimento Oncologico La MaddalenaMedicina NuclearePalermoItaly; ^16^ Dipartimento di Ematologia e Oncologia Pediatrica Istituto G. GasliniGenovaItaly; ^17^ Radiology UnitRadiology DepartmentA.R.N.A.S. Ospedali CivicoDi Cristina e BenfratelliPalermoItaly

**Keywords:** Childhood, Hodgkin Lymphoma, PET

## Abstract

The purpose of the study was to determine if abdomen/pelvis computed tomography (CT) can be safety omitted in the initial staging of a subgroup of children affected by Hodgkin Lymphoma (HL). Every participating center of A.I.E.O.P (Associazione Italiana di Ematologia ed Oncologia Pediatrica) sent local staging reports of 18F‐fluorodeoxyglucose positron emission tomography (PET) and abdominal ultrasound (US) along with digital images of staging abdomen/pelvis CT to the investigation center where the CT scans were evaluated by an experienced pediatric radiologist. The local radiologist who performed the US was unaware of local CT and PET reports (both carried out after US), and the reviewer radiologist examining the CT images was unaware of local US, PET and CT reports. A new abdominal staging of 123 patients performed on the basis of local US report, local PET report, and centralized CT report was then compared to a simpler staging based on local US and PET. No additional lesion was discovered by CT in patients with abdomen/pelvis negativity in both US and PET or isolated spleen positivity in US (or US and PET), and so it seems that in the initial staging, abdomen/pelvis CT can be safety omitted in about 1/2 to 2/3 of children diagnosed with HL.

## Introduction

Hodgkin lymphoma (HL) was the first malignancy to be definitely cured by chemotherapy and radiotherapy 1 and survival has markedly increased 2 since 1970 to more than 80%. Given that many modern approaches do not intend to increase the cure rate, but rather by limiting the use of radiotherapy 3, 4 reduce long‐term toxicities 5, there are challenging studies to develop for diagnosis and surveillance. Staging techniques have deeply changed over time: in the 1960s and 1970s, they were dominated by lymphangiogram and laparotomy, both eliminated by computed tomography (CT), a technique delivering high ionizing radiation doses 6. Subsequently there was the expansion of nuclear medicine, firstly with gallium scan, and more recently with 18F‐fluorodeoxyglucose positron emission tomography (PET), that is now firmly established as front‐line technique 7. Many trials are using interim PET after two cycles of chemotherapy to address which patients need therapy intensification, or conversely reduction, and how many patients can avoid radiotherapy. Furthermore, the question has been raised as to whether it is possible not only to reduce the radiation exposure for treatment (that is radiotherapy) but also for surveillance (that is above all surveillance CT), with some initial studies showing an overadministration of surveillance CT 8. We decided to verify if there was also an overadministration of staging CT in pediatric HL after an informal analysis among some A.I.E.O.P (Associazione Italiana di Oncologia ed Ematologia Pediatrica) Centers had not reported any cases of abdominal and pelvic localizations identified by abdomen/pelvis CT in patients with a contemporary negativity of PET and abdominal Ultrasound (US). Since it is well known that the measurement accuracy of US is inferior to CT 9, a randomized trial would be addressed as not ethical, and so we organized a complex study with a centralized revision of CT images.

## Materials

The study was designed by the HL working group of A.I.E.O.P. (Associazione Italiana di Onco‐Ematologia Pediatrica) and approved by the ethics committee of the investigation center (Pediatric Onco‐Hematology, Palermo, Italy). Informed consent for the collection of clinical data was obtained from the parents or the legal guardians according to the Helsinki declaration at the enrollment in A.I.E.O.P trial LH2004 for pediatric HL. Initially, the concordance among abdomen/pelvis ultrasound (all systems equipped with multifrequency ultrasound transducer probes), CT (at least 16‐channel multidetector CT), and PET (not PET/CT and always performed with intravenous contrast) was evaluated; to avoid the possible influence of previous radiological reports, it was necessary that the radiologist analyzing CT images was not aware of the US report, and the radiologist performing US was not aware of the CT report. To achieve this goal, every participating center sent digital images of staging CT along with staging US and PET reports of at least 80% of patients enrolled from January 1, 2008 to December 31, 2012: in this period, 512 children were prospectively enrolled in A.I.E.O.P trial LH2004. The local CT images were evaluated (centralized CT) by one expert radiologist, and cases with US performed after local CT were excluded. Consequently, only patients in whom the investigations were performed locally in the following order were analyzed: US, CT, PET. Hence, the local radiologist who performed the US did not know the local CT and PET reports (both carried out later), and the reviewer radiologist examining the CT images did not know local US, CT, and PET reports. In case of doubtful PET report, PET imaging was centrally reviewed by an expert nuclear physician (unaware of any report) on a case‐by‐case basis. Initially, a comparison between US/PET and US/PET/CT accuracy in the evaluation of any abdomen/pelvis site was done; later, a comparison between the accuracy of the Test Staging (TS) (complete abdomen/pelvis evaluation based on local US + local PET) and the Final Staging (FS) (complete abdomen/pelvis evaluation based on local US + local PET + centralized CT) was performed. The definition in US, PET, and CT of any site as involved was based on criteria adopted in the current Euronet‐PHL‐C1 study 10 but modified since in this trial, where PET is mandatory, abdomen/pelvis evaluation has to be performed with CT or Magnetic Resonance Imaging (MRI) plus liver/spleen US (not abdomen/pelvis US). In all US and CT measurements, the largest diameter, transverse, or craniocaudal, was considered. In PET, the Deauville criteria 11 were used. Briefly:

Lymph nodes

1. US/PET evaluation. In US, a lymph node (LN) or a LN conglomerate was considered not involved (negative) if its largest diameter was <1 cm, involved (positive) if it was >2 cm, and doubtful if it was 1–2 cm. Neither vascularity nor nodal architecture was included for determining US involvement. Since it is well known that the measurement accuracy of US is inferior to CT 11, US/PET evaluation was considered positive if positive in both US and PET and negative if negative in both US and PET: in all other cases, it was considered doubtful and in need of CT for definitive assessment.
2. US/PET/CT evaluation. In CT, a LN or LN conglomerate was considered not involved (negative) independent of the PET result if the largest diameter was <1 cm and involved (positive) if it was >2 cm. In the case of a diameter of 1–2 cm, the LN was classified as doubtful and considered involved if PET was positive or doubtful. Generally, in the case of discrepancy between CT and US, the CT evaluation was considered dominant, but cases of “strange” discordance (i.e., concordant PET/US positivity with CT negativity) were carefully re‐evaluated and considered positive if PET and US positivity was clearly confirmed.
3. In PET, Deauville 1–2 LNs were classified as negative, Deauville 4–5 LNs as positive, and Deauville 3 LNs as doubtful.

Spleen and liver

1. In CT and US, isolated enlargements of spleen and/or liver were not considered as involvement (negative).
2. Diffuse infiltration and focal splenic changes in US (hypo‐echoic nodular lesions) or CT (hypo‐enhancing nodules) were considered involved (positive). Every US or CT lesion atypical of tumors was considered doubtful: multiple doubtful tumor suspicious US changes were considered involved—independent of the PET and CT result.
3. In US or CT, diffuse infiltration and focal liver changes were considered involvement (positive). Every US or CT lesion atypical of tumors was considered doubtful, and multiple doubtful tumor suspicious US changes were considered involved—independent of the PET and CT result.
4. In PET, solitary or multiple splenic areas of intense radiotracer accumulation were considered lymphomatous infiltration (positive): a FDG‐avid spleen without clear focal changes was considered doubtful.
5. In PET, foci of abnormal liver FDG uptake with SUV higher than those of the surrounding parenchyma were considered involvement (positive): every suspected lesion without clear focal change was considered doubtful.
6. In the case of positive/doubtful PET associated with negative US, liver and/or spleen were considered involved only in the case of positive/doubtful CT.
7. In the case of US/PET negativity, liver and/or spleen were considered not involved even in the case of positive/doubtful CT.

Tables 1 and 2 summarize the US/PET evaluation criteria of LNs, spleen, and liver (no other abdominal/pelvic site was affected): any single site could be positive (involved), negative (not involved), or doubtful (cases where CT was mandatory to assess the involvement). Table 3 summarizes the possible results of TS: in all patients in whom US/PET evaluation was doubtful in at least one site, the TS was classified as doubtful. Centralized CT was classified as following:

**Table 1:** US/PET evaluation of lymph nodes

Lymph nodes		
US	PET	US/PET evaluation
Negative	Negative	Negative
Positive	Positive	Positive
Positive	Negative	Doubtful
Negative	Positive	Doubtful
Doubtful	Positive or Doubtful or Negative	Doubtful
Positive or Negative	Doubtful	Doubtful

PET, positron emission tomography; US, ultrasound.

**Table 2:** US/PET evaluation of spleen/liver

Spleen or Liver		
US	PET	US/PET evaluation
Negative	Negative	Negative
Negative	Positive or Doubtful	Doubtful
Doubtful or Positive	Positive or Doubtful or Negative	Positive

PET, positron emission tomography; US, ultrasound.

**Table 3:** Test staging

US/PET evaluation	Test staging
Negative in lymph nodes and spleen/liver	Negative
Positive in at least one site	Positive
Doubtful in at least one site (and no positive site)	Doubtful

PET, positron emission tomography; US, ultrasound.

1 Confounding*,* if showing:
  - Doubtful or positive LNs not confirmed as positive in the FS
  - Negative LNs not confirmed as negative in the FS
  - Doubtful or positive spleen/liver not confirmed as positive in the FS
  - Negative spleen/liver not confirmed as negative in the FS.
2 Useful*,* if:
  - CT was required for FS. For example, PET negativity in LNs doubtful in US (doubtful LNs in TS) with CT confirming diameter <2 cm (negative LNs in FS)
  - FS, if compared to TS, was modified by CT results. For example, US/PET negativity in abdominal/pelvic LNs (negative LNs in TS) with CT showing diameter >2 cm (positive LNs in FS)
3 Useless if CT was not classified as useful or confounding and the FS was identical to the TS.

In the case of CT useful for the assessment of one site (i.e., LNs) and confounding /useless for another site (i.e., spleen), the CT was finally classified as useful.

## Results

Data from 133 patients treated in 14 A.I.E.O.P centers were received. Ten cases with US performed after CT were excluded, so the analysis was performed on 123 patients: 53 female patients (43.0%) and 70 males (56.9%) with a median age at diagnosis of 13.8 years.

In LNs, the concordance between US and CT was 73.9% (91 patients: 9 positive, 79 negative and 3 doubtful). PET was positive in all three patients with LNs concordantly doubtful at US and CT. In the field of LN evaluation, there were four false‐negative USs and four false‐negative CTs (see below). In LNs, PET was positive (always with high SUV) or negative, with only one Deauville 3 doubtful LN.

In spleen, the US/CT concordance was 86.2% (106 patients: 21 positive and 85 negative). PET confirmed positivity in 15/21 of US/CT positive patients; among the 85 US/CT negative patients, there was one doubtful and one positive PET (without changing the final evaluation of negative spleen). In liver, the discordance between US and CT was restricted to one patient, positive in US and PET but negative at CT evaluation. Spleen and liver PET were positive or negative, with only two doubtful splenic cases (radiotracer‐avid spleen without focal changes).

There were 71/123 patients with a negative TS: no new lesion was diagnosed by CT. There were 27/123 patients with a positive TS: all were confirmed by CT and no site was added or withdrawn.

In 25/123 patients, the TS was doubtful: in 21 patients (84%) after CT evaluation the FS was positive, and in 4 (16%) negative. According to the study methods, in all doubtful TS cases, at least one technique (US or PET) was doubtful or positive, even though, among various TS possibilities, there are clear differences in terms of original probability of LN involvement: for example, between a US doubtful/PET negative patient and a US doubtful/PET positive patient, the latter has much more probability of becoming positive at FS. In any case if we consider doubtful TS cases as positive, an analysis in terms of sensitivity, specificity, positive predictive value (PPV), and negative predictive value (NPV) can be performed. The results are as follows:

1. Sensitivity: 1.00 (Confidence Interval (CI): 0.89–1.00)
2. Specificity: 0.95 (CI: 0.87–0.99)
3. PPV: 0.92 (CI: 0.81–0.98)
4. NPV: 1.00 (CI: 0.93–1.00)

**Figure 1:**
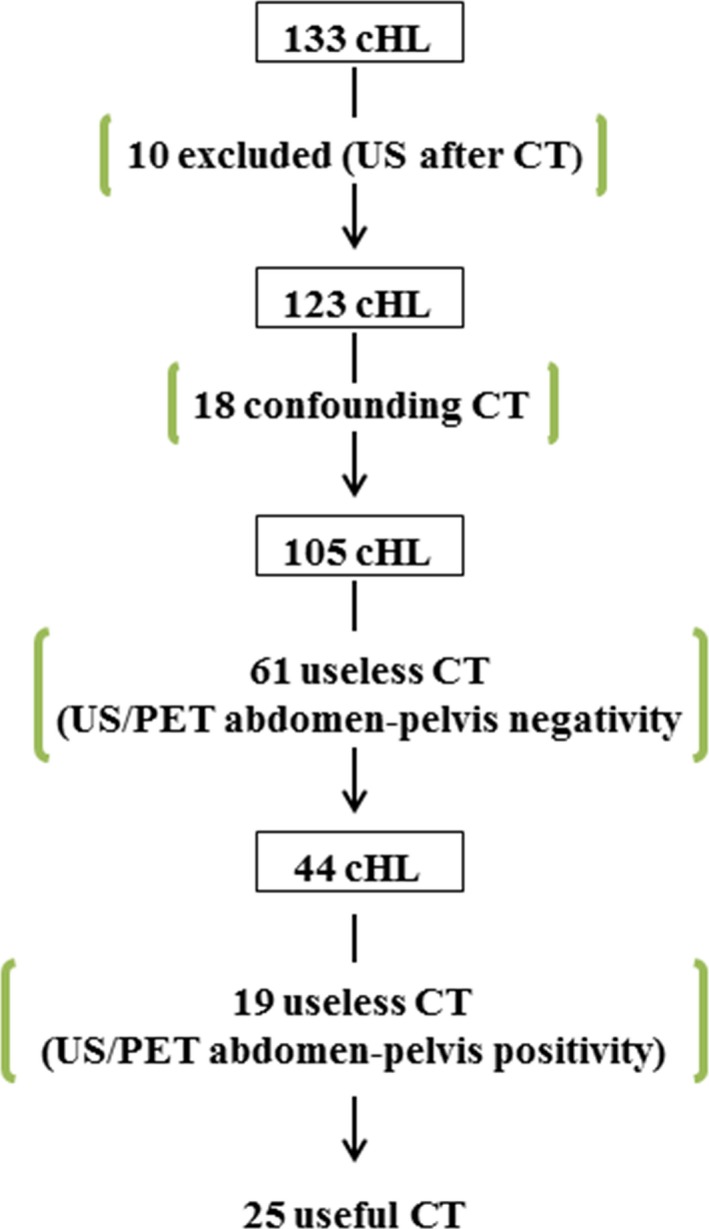
Flow chart of CT value.

Concerning CT value (Fig. 1), in 18/123 patients, CT was potentially confounding (Table 4): in 11 (lines A–C) CT addressed doubtful LNs classified as negative by US and PET, and in 1 (line D) there was also a CT false‐negative spleen. In two patients (lines H–I), there was a “doubtful” CT spleen finally assessed as a false positive. In the last four patients, CT was confounding exclusively (lines E–F) or also (line G) due to its negativity in LNs that were doubtful in US and positive in PET (with high SUV).

**Table 4:** Confounding CT

		Lymph nodes			Spleen			Liver			Final staging
LINE	Pt	US	PET	CT	US	PET	CT	US	PET	CT	
A	9	−	−	+/−	−	−	−	−	−	−	All negative
B	1	−	−	+/−	+	+	+	−	−	−	Spleen positive LN/Liver negative
C	1	−	−	+/−	+	+/−	+	−	−	−	Spleen positive LN/Liver negative
D	1	−	−	+/−	+	+	−	−	−	−	Spleen positive LN/Liver negative
E	1	+/−	+	−	+	+	+	−	−	−	LN/Spleen positiveLiver negative
F	1	+/−	+	−	+	+	+	−	−	−	LN/Spleen positiveLiver negative
G	2	+/	+	−	+	+	−	−	−	−	LN/Spleen positiveLiver negative
H	1	−	−	−	−	−	+/−	−	−	−	All negative
I	1	+	+	+	−	−	+/−	−	−	−	LN positive Spleen/Liver negative

PET, positron emission tomography; Pt, number of patients; LN, lymph nodes; LN +/− in US/CT: lymph nodes of 1–2 cm diameter; Spleen +/− in PET or CT:suspected positivity.

In 80/123 patients, CT could be considered useless (Table 5) because it did not modify any assessment of site involvement: in 61 (line A) it was negative and concordant with US/PET, in 12 (lines B–G) it was positive and concordant with US/PET, and in 7 (lines H–K) there was US spleen positivity (sufficient to establish organ involvement).

**Table 5:** Useless CT

		nodes			Spleen			Liver			Final staging
Line	Pt	US	PET	CT	US	PET	CT	US	PET	CT	
A	61	−	−	−	−	−	−	−	−	−	All negative
B	5	+	+	+	−	−	−	−	−	−	LN positiveSpleen‐liver negative
C	1	+	+	+/−	−	−	−	−	−	−	LN positiveSpleen‐liver negative
D	3	−	−	−	+	+	+	−	−	−	Spleen positiveLN‐liver negative
E	1	+	+	+	+	+	+	−	−	−	LN‐spleen positiveLiver negative
F	1	+	+	+/−	+	+	+	−	−	−	LN‐spleen positiveLiver negative
G	1	+	+	+	+	+	+	+	+	+	LN‐spleen‐liver positive
H	1	+	+	+	+	−	−	−	−	−	LN‐spleen positiveLiver negative
J	5	−	−	−	+	−	+	−	−	−	Spleen positiveLN‐liver negative
K	1	−	−	−	+	−	+/−	−	−	−	Spleen positiveLN‐liver negative

PET, positron emission tomography; US, ultrasound; Pt, number of patients; LN, lymph nodes; LN +/− in CT: lymph nodes of 1–2 cm diameter; Spleen +/− in CT:suspected positivity.

In 25/123 children (those with doubtful TS), CT was useful (Table 6): in 6 (lines A–D) for assessment of spleen involvement, in 17 (lines E–Q) for assessment of LN involvement, and in 2 (line R) for both.

**Table 6:** Useful CT (Doubtful TS)

LINE		Lymph nodes			Spleen			Liver			
	Pt	US	PET	CT	US	PET	CT	US	PET	CT	Final staging
A	1	−	−	−	−	+	−	−	−	−	All negative
B	1	−	−	−	−	+/−	−	−	−	−	All negative
C	3	−	−	−	−	+	+	−	−	−	Spleen positiveLN–liver negative
D^a^	1	−	−	+/−	−	+	+	−	−	−	Spleen positiveLN–liver negative
E	1	+	−	+/−	−	−	−	−	−	−	All negative
F	1	+/−	−	−	−	−	−	−	−	−	All negative
G	1	−	+/−	−	+	+	+	−	−	−	Spleen positiveLN–liver negative
H	1	+/−	−	−	+	+	+	−	−	−	Spleen positiveLN–liver negative
I^a^	1	−	+	−	+	+	−	−	−	−	Spleen positiveLN–liver negative
J^a^	2	+/−	−	−	+	+	−	−	−	−	Spleen positiveLN–liver negative
K	1	+/−	+	+	−	−	−	−	−	−	LN positiveSpleen‐liver negative
L	1	−	+	+	−	−	−	−	−	−	LN positiveSpleen‐liver negative
M	2	−	+	+/−	−	−	−	−	−	−	LN positiveSpleen‐liver negative
N	3	+/−	+	+/−	+	+	+	−	−	−	LN‐spleen positiveLiver negative
O	1	+/−	+	+	+	+	+	−	−	−	LN‐spleen positiveLiver negative
P	1	−	+	+/−	−	−	−	+	+	+	LN‐liver positiveSpleen negative
Q^a^	1	−	+	−	+	+	−	+	+	−	Spleen‐liver positiveLN negative
R	2	−	+	+/−	−	+	+	−	−	−	LN‐spleen positiveLiver negative

PET +/−: suspected positivity in LN or spleen.

US, ultrasound; TS, Test Staging; PET, positron emission tomography; Pt, number of patients; LN, lymph nodes; LN +/− in US/CT: lymph nodes of 1–2 cm diameter.

a Patients in whom CT was useful about lymph nodes evaluation but useless/confounding about spleen and/or liver evaluation or vice versa.

In spleen, there were two cases with isolated doubtful CT (Table 4, lines H–I) and no case of isolated CT positivity. Remarkably, 12/123 patients (9.7%) (Table 4: lines B–D; Table 5: line D, J, K) were US or US/PET spleen positive (with or without CT positivity) in a context of concordant US/PET/CT negativity in LNs.

In LNs, there were four cases of false‐negative US (Table 6: lines L, M and P) and four cases of false‐negative CT (Table 4: lines E–G). In these four patients with false‐negative CT, the only ones in whom US evaluation of LNs was considered superior to CT, the US/PET positivity was always related to splenic hilum LNs. The local CT report was examined in three out of four and it was negative too. Furthermore the reviewer radiologist, after being informed of the discrepancy with US/PET, reanalyzed the CT images and recognized in two out of three the presence of doubtful LNs not identified in his first report: in the last one, which was US/CT/PET positive in spleen, US reported a 1.8 cm LN with a high SUV PET.

## Discussion

Cancer incidence in childhood has been increasing over time: in Europe, the age‐standardized incidence rate per million increased from 147 in the 1970s to 190 in the 1990s 12, and in Italy, the incidence rose by an average of 2% per year from 1998 to 2002 13. The use of CT and PET has also been increasing significantly over time 14. Both techniques deliver high ionizing radiation 15 and it has been postulated that 2% of future cancers could be a consequence of current CT rates 16. According to a study on 1656 women who underwent chest CT, a person studied two or more times doubles her 10‐year risk of breast cancer 17. Abdominal CT, whose average radiation dose is 8–11 mSv 18, also increases cancer risk 19. Furthermore, children are more sensitive to radiation: youth allows many years for cancer to develop; HL occurs most frequently in an age (10–19 years) which is 10 times more sensitive to radiation risk than adults 20 and HL is at particularly high risk of secondary neoplasm (SN) 21, 22. In the U.S.A. on the basis of the yearly rate of CT, it has been postulated that a remarkable number of children will die from CT‐induced neoplasms 15, 23, and in a retrospective study in young people, an association between CT and risk of leukemia and brain tumor was found 24. Another paper 25 recently drew attention to the increased risk in CT exposed children, with an incidence after pelvis/abdomen CT particularly elevated for leukemia, myelodysplastic syndrome, and soft tissue neoplasms.

The potential risk linked to overuse of diagnostic radiation has recently inspired two different international campaigns, “Image Gently” and “Eurosafe”, both aimed at understanding what are the most careful practices in using ionizing radiation. Some recent studies have addressed a simple algorithm including US that can help diagnose appendicitis in children and reduce CT scan 26, or that US can be preferred to CT in suspected nephrolithiasis without impacting outcome or complications 27. Furthermore, it was shown 8, 28, 29 that, in the field of surveillance after HL, the vast majority of HL relapses are identified based on clinical or laboratory findings and that survival is not affected by the modality of detection; consequently in 2013, the American Society of Hematology 30, as member of the Choosing Wisely project, included “*surveillance CT scans in asymptomatic patients after curative‐intent treatment for aggressive lymphoma*” among tests and treatments not well supported by evidence. In 2013/14, the American Academy of Pediatrics 31 identified some conditions of possible overuse of CT among tests that should be limited. So, based on the evidence that the majority of HL presentation is in the mediastinum and/or in a superficial lymphadenopathy (abdomen/pelvis disease is present in 35.0% of patients enrolled in the LH2004 trial and in 39.0% in the present series), we decided to also explore the possibility of reducing CT scans during the staging.

In our analysis, there were 71 patients with a complete concordant US/PET negativity (lines A and H of Table 4 and line A of Table 5): CT did not contribute in any of them. In all 27 patients with a positive TS, the FS turned out to be positive too. Interestingly, it seems that the accuracy of US is higher than CT in detecting splenic hilum LNs: the poor fat cleavage in left hypochondrium structures and in axial CT sections the overlapping of parts of pancreas tail, spleen, and splenic vessels may be the cause of failed recognition of these LNs. Surprisingly, in the present analysis, there is the same number (4) of US false‐negative LNs. Probably three‐plane reconstruction can help in better defining lymph nodes.

Finally, there were 25 patients, all with doubtful TS (and so with some kind of “positivity” at US/PET staging), where CT was necessary to determine abdomen/pelvis as involved in HL or not.

In the LH04 trial, the Lugano update of the Ann Arbor system 32 where PET and CT are formally incorporated into standard staging, was used at diagnosis: this group of 25 patients is the only one where abdominal CT was necessary to define the exact staging, with the consequent treatment decision.

The primary limitation of this study, justified by the complex organization of the imaging review, is the number of enrolled patients. Nonetheless, we think that the statistical results are consistent, and that according to our data, abdomen/pelvis CT could be safely omitted in cases with:

1. Concordant abdomen/pelvis US/PET negativity. In our study, they were 57.7%.
2. Concordant US/PET positivity or isolated US positivity in spleen associated with concordant US/PET negativity in LNs. In our study, they were 9.7%.

With this approach CT could be omitted in about 2/3 of patients (67.4%). Furthermore what seems clear, according to our data, is that with the strictest approach, it is possible to omit CT at least in PET/US negative patients: this is a group (57.7%) where CT never modifies the FS, but also in many cases raises doubts about later unconfirmed sites of disease. Such an approach is certainly supported in our analysis by the 100% NPV, with a very narrow 95% CI (93–100%). CT remains useful in whatever type of LN positivity in PET and/or US, even if clear and concordant, since CT can better define the exact site of LN involvement and assist the choice as to radiotherapy planning.

Avoiding abdomen/pelvis CT would have some positive effects in patients in whom it is deemed possible:

1. Reducing radiation exposure.
2. Cutting possible side effects and adverse/allergic reactions of contrast materials and of general anesthesia (in the minority of patients needing it) by reducing time of the examination and drug doses.
3. Cutting the costs: in Italy, the average cost of abdomen/pelvis CT and abdomen/pelvis US is 190–200 and 60–70 Euros, respectively. In the U.S., the cost is much higher.

All these advantages would be present even compared to an initial staging performed through combined PET/CT (where usually a CT scan with iodine contrast media is performed after PET) or, apart from the radiation risk, through chest CT and abdomen/pelvis MRI, which is actually a much more complicated approach.

In cancer, the clinical benefits of accurate staging far outweigh the increased risk of SN, but since along with an elevated risk of SN, HL patients present abdominal involvement in only about 1/3 of patients, we believe it is time for an evidence‐based assessment of the impact of staging abdomen/pelvis CT in the care plan. Even though there is need for further study, our work indicates that CT can be safety omitted in at least half of patients. A reduction of CT use should provide some definite benefits, and probably induce a slightly advantageous reduction in SN. Finally, this new approach could accomplish a significant reduction in financial costs.

## Conflict of Interest

The authors declare that they have no conflicts of interest.
